# Extruded Nucleoli of Human Dental Pulp Cells

**DOI:** 10.3390/medicina58020260

**Published:** 2022-02-10

**Authors:** Mugurel Constantin Rusu, Alexandra Diana Vrapciu, Mihnea Ioan Nicolescu, Mihai Dragomir Stoenescu, Adelina Maria Jianu, Rodica Lighezan, Roxana Oancea, Vasile Sorin Mănoiu, Sorin Hostiuc

**Affiliations:** 1 Division of Anatomy, Faculty of Dental Medicine, “Carol Davila” University of Medicine and Pharmacy, 050474 Bucharest, Romania; mugurel.rusu@umfcd.ro (M.C.R.); alexandra.vrapciu@umfcd.ro (A.D.V.); 2 Division of Histology, Faculty of Dental Medicine, “Carol Davila” University of Medicine and Pharmacy, 050474 Bucharest, Romania; 3 Laboratory of Radiobiology, “Victor Babeș” National Institute of Pathology, 050096 Bucharest, Romania; 4 Research Department, “Dr. Carol Davila” Central Military Emergency Hospital, 010825 Bucharest, Romania; dragomir.stoenescu@drd.umfcd.ro; 5 Department of Anatomy, Faculty of Medicine, “Victor Babeş” University of Medicine and Pharmacy, 300041 Timişoara, Romania; 6 Department of Histology, Faculty of Medicine, “Victor Babeş” University of Medicine and Pharmacy, 300041 Timişoara, Romania; lighezan.rodica@umft.ro; 7 Department of Preventive and Community Dentistry, Faculty of Dental Medicine, “Victor Babeş” University of Medicine and Pharmacy, 300041 Timişoara, Romania; oancea.roxana@umft.ro; 8 Department of Cellular and Molecular Biology, National Institute of Research and Development for Biological Sciences, 060031 Bucharest, Romania; spagiricus@gmail.com; 9 Division of Legal Medicine, Faculty of Dental Medicine, “Carol Davila” University of Medicine and Pharmacy, 020021 Bucharest, Romania; sorin.hostiuc@umfcd.ro

**Keywords:** dental pulp, stem niche, transmission electron microscopy, nucleolus, nucleolar channel system

## Abstract

*Background and Objectives*: The dental pulp stem cells are highly proliferative and can differentiate into various cell types, including endothelial cells. We aimed to evaluate the ultrastructural characteristics of the human dental pulp cells of the permanent frontal teeth. *Materials and Methods*: Human adult bioptic dental pulp was collected from n = 10 healthy frontal teeth of five adult patients, prior to prosthetic treatments for aesthetic purposes. Tissues were examined under transmission electron microscopy. *Results*: We identified cells with a peculiar trait: giant nucleoli resembling intranuclear endoplasmic reticulum, which mimicked extrusion towards the cytoplasm. These were either partly embedded within the nuclei, the case in which their adnuclear side was coated by marginal heterochromatin and the abnuclear side was coated by a thin rim of ribosomes, or were apparently isolated from the nuclei, while still being covered by ribosomes. *Conclusions*: Similar electron microscopy features were previously reported in the human endometrium, as nucleolar channel system; or R-Rings induced by Nopp140. To our knowledge, this is the first report of extruded nucleolar structure in the dental pulp. Moreover, the aspect of giant extruded nucleoli was not previously reported in any human cell type, although similar evidence was gathered in other species as well as in plants.

## 1. Introduction

The dental pulp (DP), entrapped within the “sealed niche” of the pulp chamber, is particularly interesting in regenerative medicine due to its accessibility and differentiation potential [1,2]. There are several zones in the DP, each with its own cellular repertoire and specific role. The DP outer odontoblast layer ensures the production of dentin matrix and its subsequent calcification [3]. The cell-free zone of Weil is followed by a cell-rich zone and finally the pulp core. The core harbours various connective cell populations, including DP stem cells—a cell population derived from neural ectomesenchyme, with a high proliferative profile and immunosuppressive activity [4,5]. Besides having a multilineage potential [6], dental pulp cells may release, under hypoxic stress, proangiogenic factors [7]. However, since hematopoietic progenitors are not identifiable within the DP [8], DP stem cells can promote adult vasculogenesis by differentiating into endothelial-like cells (ECs) in the right microenvironment [7].

The human DP was rarely characterized under transmission electron microscopy (TEM) [9,10]. Prosthetic treatments in the frontal area may require endodontic treatment [11,12,13], hence we decided to evaluate under TEM the ultrastructure of healthy DP harvested from the frontal teeth.

We aimed thus to evaluate the ultrastructural characteristics of the human dental pulp, mainly the DP cells exhibiting (pre)mitotic aspects or peculiar features. After unveiling the particular nucleolar features of some of the DP cells we encountered, we decided to report this in the present study, considering a feature that might represent a trait of some intermediate cells, such as transit-amplifying DP cells.

## 2. Materials and Methods

Human adult biopsy material (dental pulp) was collected from n = 10 healthy frontal teeth (incisors and canines) of 5 adult patients (3 females and 2 males; mean age 37.5 years), prior to prosthetic treatments for aesthetic purposes. Informed consent for use of the biopsy material for research purposes was obtained from the patients. The study was conducted according to the guidelines of the Declaration of Helsinki and approved by the Ethics Committee of the “Dr. Carol Davila” Central Military Emergency Hospital (No. 372 on 18 March 2020).

Tissue samples were prepared for TEM as described previously [14]. Small tissue fragments were prefixed in fresh ice-cold 4% glutaraldehyde in sodium cacodylate buffer (pH 7.4) for 4 h at 4 °C. After fixation, the tissues were washed 6 times with 0.05 M sodium cacodylate buffer (pH 7.4) at 4 °C, subsequently postfixed in 2% osmium tetroxide in 0.1 M sodium cacodylate at room temperature for 2.5 h, stained en bloc with 0.5% aqueous uranyl acetate overnight at 4 °C and washed with 0.05 M sodium cacodylate buffer. After dehydration in a graded ethanol series and infiltration with propylene oxide, the specimens were embedded in glycidyl ether (Epon 812-equivalent) and finally polymerized at 60 °C for 48 h. Semithin sections were stained with 1% toluidine blue. Ultrathin sections (80–100 nm) were cut by using a diamond knife, collected on 200 mesh copper grids, and double counterstained with uranyl acetate followed by lead citrate. The grids were examined under a Philips electron microscope EM 208S (acceleration voltage of 80 kV), and snapshots were captured by using a Veleta video camera and the iTEM Olympus Soft Imaging System.

## 3. Results

**Figure 1:**
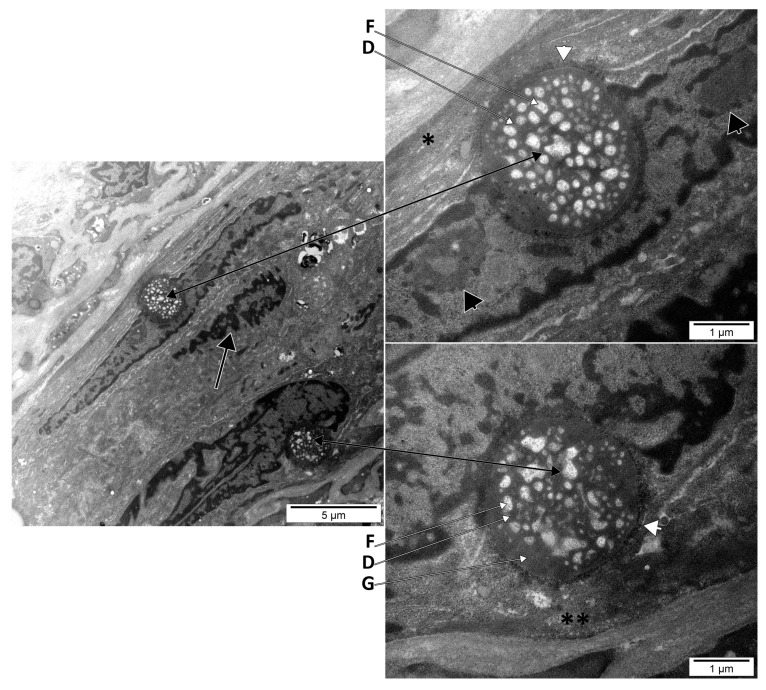
Electron micrograph of the dental pulp of an upper lateral human incisor. Giant nucleoli of two different spindle-shaped DP cells are presented in situ (**left panel**) and detailed at higher magnification in the two right panels. In the left panel, we identified (black arrow) a cell with similar nuclear morphology, but without any peculiar features. Each of the two depicted nucleoli consists of fibrillar centers (F), dense fibrillar components (D), and a granular component (G). On the adnuclear side, they are coated by heterochromatin, and on the cytoplasmic side, they are coated by a thin rim of (pre)ribosomes (white arrowheads). Intermediate filaments (*) and caveolae (**) are also visible in the respective cells.

We identified peculiar ultrastructural aspects of some nucleoli. Mature nucleoli with homogenous morphologies were identified in spindle-shaped cells (Figure 1). They were most likely linked to the nucleolar organizer regions (NORs) of those nuclei, either central or polar. Interestingly, those nucleoli were of comparable sizes and were partly embedded in the nucleus and partly extruded in the cytoplasm. They consisted of fibrillar centres, dense fibrillar components, and the granular component; on the adnuclear side, they were coated by heterochromatin, while on the abnuclear (cytoplasmic) side they were coated by a thin rim of (pre)ribosomes, in direct contact with the intermediate filaments from the cytoplasm. The presence of the (pre)ribosomes helped us to consider these nucleoli as being mature, functional, and not the primitive type, which should have been larger and lacking a ribosomal coat.

**Figure 2:**
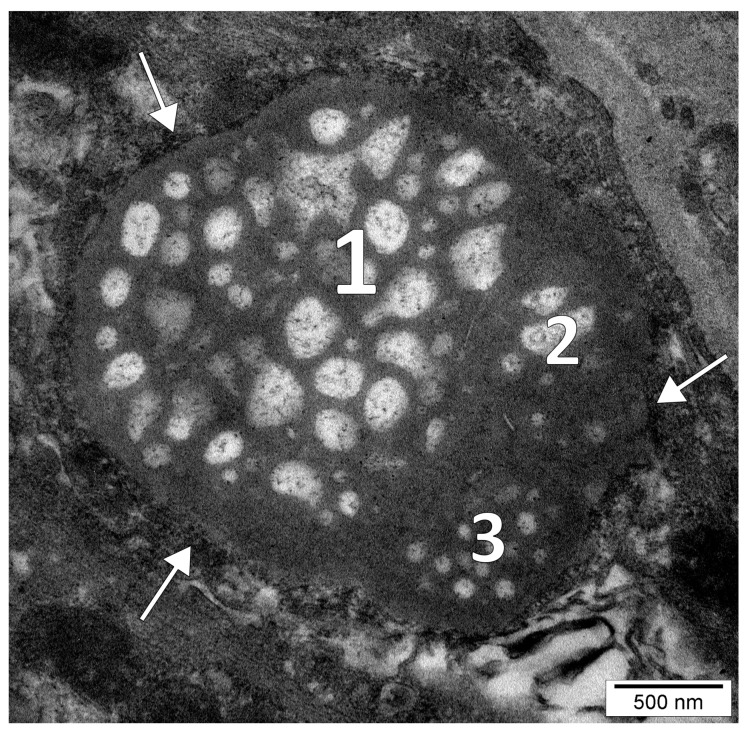
High magnification electron micrograph of the dental pulp of a human upper lateral incisor. A multilocular nucleolus is depicted consisting of three (1–3) aggregated bodies and coated by a layer of pre-ribosomes (white arrows).

A multilocular nucleolus completely coated by pre-ribosomes and was seemingly composed of three nucleolar bodies (Figure 2) which were fused together at the level of their granular components (it could not be excluded, however, a three-dimensional multilobate shape of a single nucleolus).

**Figure 3:**
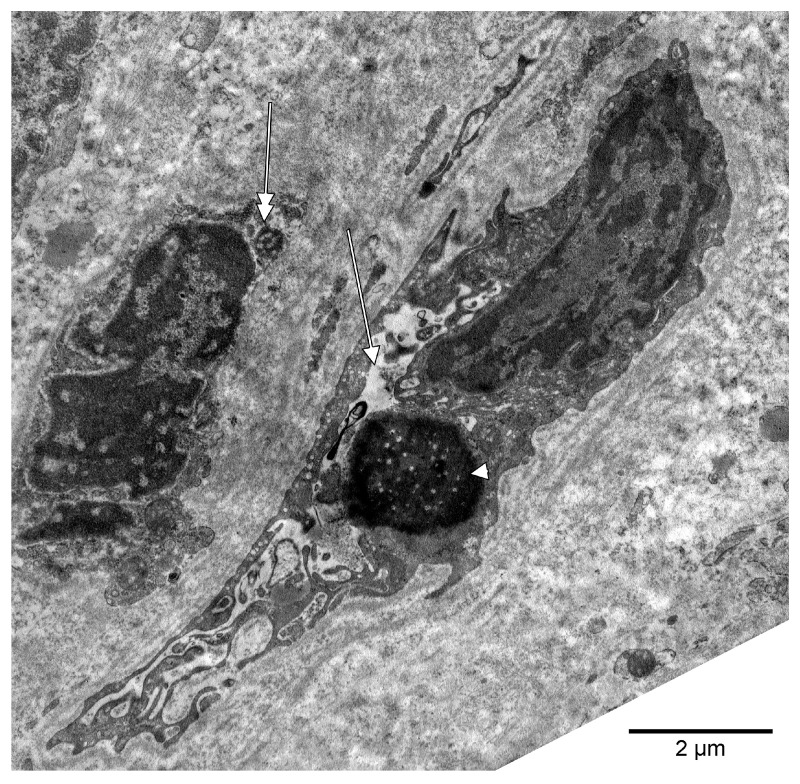
Electron micrograph of the dental pulp of a human upper canine. A possible nascent lumen (arrow) is surrounded by a putative endothelial progenitor with a giant extruded nucleolus (arrowhead). A neighbor stromal cell has also a small nucleolar-like cytoplasmic inclusion (double-headed arrow).

**Figure 4:**
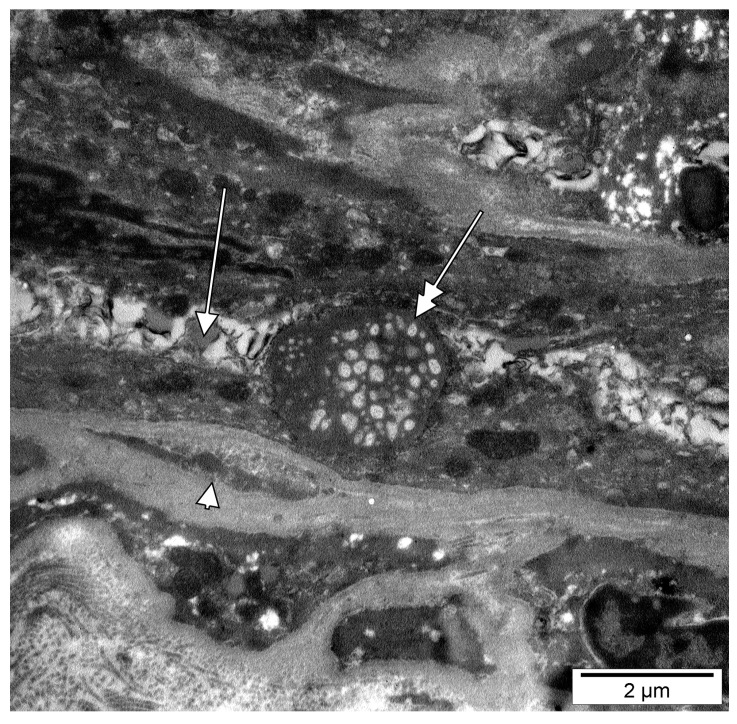
Electron micrograph of the dental pulp of a human upper lateral incisive. A giant extruded multilobate nucleolus (double-headed arrow) is associated with a lumen-acquiring (arrow) cord. A pericyte fragment (arrowhead) is closely attached to that vasculogenic cord.

**Figure 5:**
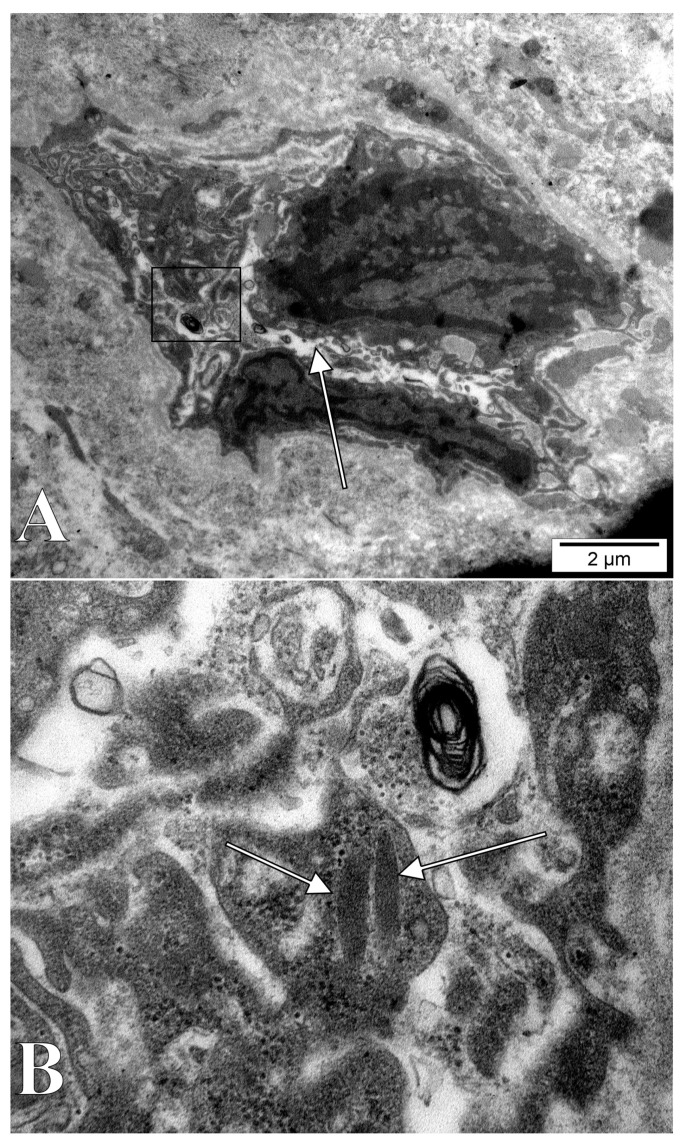
Electron micrograph of the dental pulp of a human upper canine. General view (**A**) detailed (inset) in (**B**). A lumen-acquiring cord ((**A**), arrow) is built up by Weibel-Palade bodies containing ((**B**), arrows) endothelial cells.

Dental pulp microvessels, consisting of an endothelial basal lamina, with enclosed pericytes and/or pericyte processes, were identified. The ultrastructural traits of these pericytes included plasmalemmal specializations, namely dense plaques and caveolae. In some instances, we identified cellular cords built up by poorly differentiated cells, which were apparently creating future lumina, thus we assessed them as vasculogenic (Figure 3, Figure 4 and Figure 5A). These vasculogenic cords were occasionally covered by pericytes (Figure 4). The putative endothelial progenitor cells contained giant extruded nucleoli (Figure 3 and Figure 4) and, scarcely, Weibel-Palade bodies (Figure 5B).

## 4. Discussion

### 4.1. The Nucleolar Channel System

We hereby found previously unreported structures in human tissues, which we termed giant extruded nucleoli, which seem identical in structure and size with common intranuclear nucleoli. One could diagnose these extruded nucleoli as nucleolar channel systems (NCSs). Midluteal phase endometrium consistently forms NCSs regardless of fertility status [15]. The NCS, alternatively termed nucleolar basket or nucleolar canaliculi, was identified only as a transient structure in nuclei of epithelial endometrial cells [16]. NCS are believed to be human-specific [17].

Membrane systems with a striking ultrastructural resemblance to the NCSs, termed R-rings, were induced in nuclei of tissue culture cells by the overexpression of the nucleolar protein Nopp140, consisted of several layers of tubular membrane cisternae embedded in an electron-dense matrix and were often associated with nucleoli and the nuclear envelope [17]. Such R-rings consist of *bona fide* endoplasmic reticulum but are devoid of nuclear envelope-specific structures [17].

**Figure 6:**
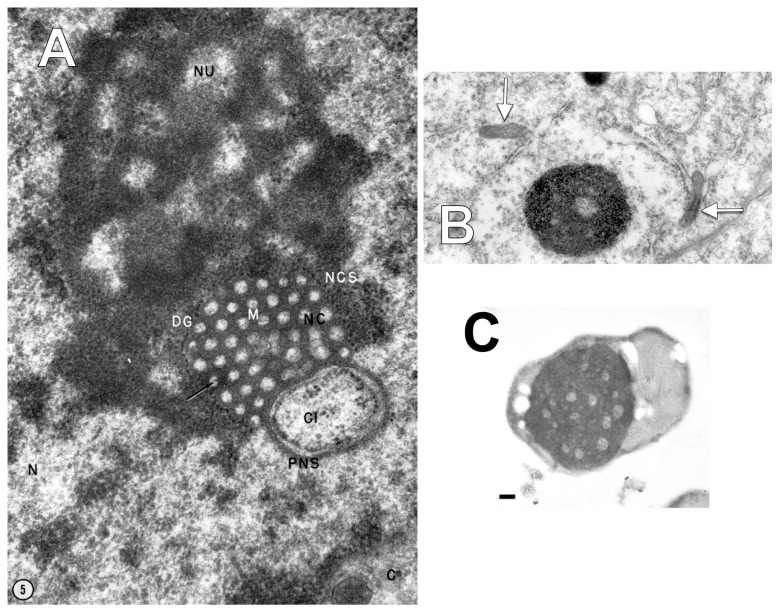
(**A**). Figure reprinted with permission from Rockefeller University Press (License Number: 4423081100494) from [18]. The author indicated (Figure 5 in the original paper): “A portion of an endometrial secretory cell nucleus (N), nucleolus (NU), and cytoplasm (C). Note that the nucleolar channel system (NCS) is located at the periphery of the nucleus. Note also that a cytoplasmic arm (CI) has pushed its way into the nucleus. The nucleolar channel system is cross sectioned showing a large number of channels (NC) in circular profile about 500- to 600-Å in diameter. Within the channels are amorphous material of low density and small granules. One granule (arrow) has a hollow central portion giving it a ring-shaped appearance. The channels are embedded in a rather dense, amorphous matrix material (M). Just peripheral to the matrix is a row of very densely staining 150-Å granules (DG). Note the difference in staining of these granules and those in the remainder of the nucleolus. Perinuclear space (PNS). X 79,000.” (**B**). Figure reprinted with permission from Springer-Verlag (License Number: 4421810018371) from [19]. The authors indicated (Figure 4 in the original paper): “A large round dense body shows even accumulation of granular particles except for several rather clear spaces. Around the body there are large clusters of ribosomes, which seem to be released from the periphery of the body. Nucleus paraventricularis. X 25,000”. One could observe the Weibel-Palade bodies (arrows) content of those cells. (**C**). Figure reprinted with permission from John Wiley and Sons (License Number: 4421800016441) from [20]. The authors indicated (Figure 5A in the original paper): “HeLa cell: actinomycin D 1 μg/mL for 20 h. A single extruded nucleolus within a cytoplasmic bleb. bar = 0.5 μm”.

Ultrastructural approaches are the only ones able to unequivocally identify NCSs [17]. Terzakis (1965) accurately documented and described the morphological possibilities of the NCS [18]. The NCS consists of matrix, dense granules, and branched and anastomosed channels, with the amorphous matrix being as dense as the most dense component of the nucleolus (Figure 6A) [18]. The shape of the NCS is variable, appearing either as a solid ellipsoid or sphere, as a thin hollow sphere, a torus, or a ring [18]. The NCS is often at the periphery of the nucleolus or separated from it [18]. It may be separated from the nuclear envelope by nucleoplasm, or it may be in contact with the inner nuclear membrane; the NCS channels being thus opened in the perinuclear space which, in turn, connects with the rough endoplasmic reticulum (RER) [18]. A third possibility for the NCS is that it directly communicates with the cytoplasm [18]. Although uncommon, the NCS evaginates from the nucleus while still being covered by the nuclear envelope [18].

However, there was no obvious evidence of nucleolus-linked NCSs, such as in the study of Terzakis (Figure 6A) and the nucleoli we found did not display the regular size and uniform repartition found in NCSs. Moreover, the lumen of the individual tubules in NCSs was found to directly communicate with the perinuclear space [18], which was not the case in our study. On the other hand, the ribosome coating we identified could represent a peculiar form of rough endoplasmic reticulum, i.e., a slightly modified NCS.

### 4.2. Intracytoplasmic Extruded Nuclear Chromatin and Nucleoli

Cassperson and Schultz (1940) discussed that “the material exchanges between nucleus and cytoplasm were studied actively in the early days of cytology” when “at the center of the discussion was the nucleolus, which in its staining properties resembled some of the cytoplasmic components, and in some cases appeared indeed to be extruded into the cytoplasm” [21]. We followed this path and found a few available studies which support the present results.

Nucleolus-like bodies were first described by Holmgren in 1899 in the cytoplasm of spinal ganglion neurons in rabbits and frogs [22], as quoted by Santolaya (1973) [23]. According to Santolaya (1973), similar structures were found in the neurons of the human spinal ganglion by Körner (1937), and in the cells of the human pineal organ by Bargmann (1943) [23,24,25]. These nucleolus-like bodies represent nucleoli extruded from the nuclei into the neuronal cytoplasm [26].

Ehret and Powers (1955) studied the nucleolar development in Paramecium Bursaria and commented that “since it has been observed that some nucleoli as small as 0.2 μm diameter are extruded, it is possible to consider enlargement of these within the cytoplasm by growth or coalescence” [27].

Shimizu and Ishii (1965) found on rat hypothalamus, using TEM, occasional nucleolus-like bodies in the cytoplasm, and considered them to be the result of nucleolar extrusion, and not artifacts [19]. By re-analyzing their images (Figure 6B) we identified bona fide Weibel-Palade bodies within those cells with cytoplasmic nucleolus-like bodies. These could have been easily overlooked, as they were described just one year before by Ewald Weibel and George Palade [28]. This observation relates the cytoplasmic nucleoli to the endothelial lineage.

Kawabata (1965) also studied the rat hypothalamus, reported nucleolus-like cytoplasmic inclusion bodies, and further discussed the probable mechanism of extrusion of nucleoli into the cytoplasm of some of the neurosecretory cells [29]. They failed to obtain a direct morphological proof of the extrusion: “*unfortunately direct morphological findings of the extrusion of the nucleolus from the nucleus were not obtained, though hundreds of sections of the hypothalamus were carefully examined*” [29]. This is not the case with our study, which showed, although static, the nuclear extrusion of cytoplasmic nucleoli.

Biggiogera et al. (1997) observed that during the apoptosis of thymocytes, nuclear ribonucleoprotein (RNP) components form clusters that are extruded into the cytoplasm [30]. The research group observed that these heterogeneous ectopic RNP-derived structures (HERDs) consist of perichromatin fibrils, interchromatin granules, perichromatin granules, and nucleolar material [30,31]. Ultrastructural evidence was further brought [20] (Figure 6C). It is thus reasonable to speculate that the presence of the extruded nucleoli in DP vasculogenic cords could relate to the lumen-acquiring phase of vasculogenic cords in which cell remodelling occurs.

Interestingly, extruded nucleoli are not a specific feature of mammalian tissues. Sparrow and Hammond (1947) documented that extranuclear chromatin have equally been described in botanical and zoological literature and brought evidence of cytoplasmic bodies with chromatin-like staining properties in the microsporocytes of several genera of plants [32]. Li et al. (2015) investigated the effects of cytogenetical alterations determined by aluminium in sunflower (*Helianthus annuus L.*) meristem cells and found that in experimental conditions, nucleolar material was extruded from the nucleus into the cytoplasm [33]. Shi et al. (2017) studied the effects of cadmium on barley cells (*Hordeum vulgare*) and found that it can have toxic effects on nucleolus and affect the expression of nucleolar proteins, leading to extrusion of nucleolar material into the cytoplasm [34]. Similarly, nucleolar extrusion was found in *Vicia faba* [35].

Nucleolin, a protein located mainly in dense fibrillar regions of the nucleolus, in addition to being present in the nucleus, is also found in the cytoplasm and plasmalemma [36,37]. Nucleolin, as well as another protein of 38 kDa (B23/No38), were commonly related only to nuclei. However, it was demonstrated that the major nucleolar proteins shuttle between the nucleus and the cytoplasm [38,39].

### 4.3. Nucleolar Extrusion in Cancer Stem Cells

The extruded nucleoli we found were much larger than the corresponding nuclei. This could suggest a nucleolar aberration, such as in malignant cells in which a disputed concept states that the “*nucleolus is much larger in proportion to the size of the nucleus in all malignant cells regardless of the type or origin of the neoplasm*” [40].

The anticarcinogenic emodin inhibits cancer cells growth, increases the mRNA and protein expression of Notch1 while significantly decreasing the mRNA and protein expression levels of VEGF [41]. Notch1 is not only localized in the membrane and cytoplasm but also in the nucleolus of cancer cells and is involved in tumor-suppressing mechanisms [41]. Activation of the Notch signalling pathway induces the downregulation of VEGF by suppressing tumorigenesis and angiogenesis [41]. Interestingly, teeth injuries activate the expression of nestin in odontoblasts and Notch in DP cells, including those cells found in blood vessel cells [42]. Therefore, further studies are mandatory to evaluate the expression of Notch in DP cells and to locate it within the cell components, including the nucleoli. Although Notch activation in cancer stem cells is driven by juxtacrine signalling between these cells and the endothelial cells in those niches [43], it looks speculative to extrapolate this mechanism to the non-malignant DP niche. 

### 4.4. Which Is the Role of the Extruded Nucleoli in the Dental Pulp?

Recently, Bhartiya commented that “*it is intriguing that in 2018 we are discussing the definition of stem cells*” [44]. This viewpoint arose from personal results as well as from the recent observation of Caplan, that MSCs derived from perivascular cells, the “*pericytes*”, and should be renamed to “*Medicinal Signalling Cells*” [44,45]. The clarifications of Caplan (2017) override the first definition of MSCs, which resulted also from his studies of human bone marrow cells [45,46]. Pericytes, consistently viewed as key players of the perivascular/periendothelial stem niche [47,48,49,50,51,52,53], are involved in bidirectional transdifferentiation: on one hand, in the adluminal flow from mesenchymal-to-endothelial cells [54,55] and reversely, in the abluminal flow from endothelial-to-mesenchymal, or to stem cells [9,56]. In these regards, transit-amplifying cells in the niches will display enough phenotypic versatility to blur an exact cell type identification. We think that these peculiar nucleoli could belong to these transit-amplifying cells, but further tests are needed to establish the functional value of the extruded nucleoli in the dental niche, as well as to identify whether such substructures are specific or not to this niche.

It has been reported that in plants, the extruded nucleoli might represent a reaction to hypoxia [57] or metal exposure [58]. In our human DP samples, the presence of extruded nucleoli might be a result of DP cells reacting to the metal from the files used to extract the DP from the tooth by the dentist.

## 5. Limitations of the Study

We have only studied a limited number of patients since we aimed this as a pilot study to report a peculiar aspect in some DP cells.

We have presented electron micrographs of peculiar nucleoli morphology that resemble the intranuclear membrane proliferation previously described only in postovulation human endometrium. However, more detailed investigations are needed to establish the exact role of the giant extruded nucleoli in the dental pulp.

## Data Availability

The data presented in this study are available on request from the corresponding author.
